# Conserved Arginine of the Potyviridae Viral Genome-Linked Proteins (VPg) as a Key Determinant for eIF4E Binding

**DOI:** 10.3390/ijms27073280

**Published:** 2026-04-04

**Authors:** Victoria V. Kolesnikova, Ekaterina Yu. Nikonova, Stanislav V. Nikonov, Alisa O. Mikhaylina, Ilia B. Simis, Vladimir V. Andreitsev, Phat T. Do, Oleg S. Nikonov

**Affiliations:** 1 Institute of Protein Research, Russian Academy of Sciences, Pushchino 142290, Moscow Region, Russia; 2 Institute of Biology, Vietnam Academy of Science and Technology, Hanoi 100000, Vietnam; dtphat@ibt.ac.vn

**Keywords:** Potyviridae, MSA, molecular modelling, molecular dynamics, eIF4E, VPg

## Abstract

Plant viruses from the Potyviridae family have a significant impact on crop productivity worldwide. We conducted a bioinformatic analysis of the VPg sequences from several members of the Potyviridae family. All analyzed primary structures of VPg contain an invariant arginine, which, according to the model we proposed earlier, is located in the functionally important α1–α2 hairpin of the viral protein and forms a recognition contact during the formation of its complex with the eIF4E host cell. Among the amino acid mutations observed in the sequences of VPg PVY, we separately considered those associated with adaptation to the host plant. Several strain-specific mutations were identified, the functional roles of which are currently unclear. For each of the Potyviridae species considered, a consensus VPg sequence was determined. 3D-models of the corresponding proteins were constructed by de novo molecular modelling using the consensus amino acid sequences. Cross-comparative analysis of the theoretical models and the experimental VPg PVY structure obtained by NMR showed that all these proteins share a high degree of structural homology and contain the conserved arginine within the α1–α2 hairpin. However, the spatial position of this arginine may vary across models, which apparently reflects species-specific differences in the VPg recognition module.

## 1. Introduction

The Potyviridae family comprises several genera, representatives of which affect economically important crops. These include wheat yellow mosaic virus (WYMV) from the genus Bymovirus, cassava brown streak virus (CBSV) from the genus Ipomovirus, plum pox virus (PPV), and potato virus Y (PVY) from the genus Potyvirus. The latter is one of the most studied and harmful members of the Potyviridae family [1,2,3]. PVY is represented by a large variety of strains, most of which are recombinant [4,5,6,7,8]. The main host plant of this virus is potato, though it is also capable of infecting other members of the Solanaceae family.

One of the most important stages in the life cycle of viruses is the initiation of translation, when the ss(+)RNA of potyviruses is translated by the protein-synthesizing machinery of the host cell. The mechanism of translation initiation in potyviruses is poorly studied. It is believed that, in representatives of this virus family, translation initiation follows a path that almost completely corresponds to the canonical cap-dependent mechanism. The key difference is that the cap analogue in this case is a small VPg protein (viral genome-linked protein) covalently bound to the 5′ end of the viral RNA, which interacts with the eukaryotic translation initiation factor 4E (eIF4E) of the host cell [9,10,11,12,13]. According to experimental data, VPg plays a crucial role in pathogenesis. It has been shown that if this protein loses the ability to interact with host eIF4E, the virus fails to infect the plant [12,14,15,16,17,18]. An obvious strategy for combating viral infection would be to disable the genes encoding susceptibility factors, namely the proteins of the eIF4E family. However, a knockout or knockdown of even part of the genes encoding these proteins can significantly affect plant viability. Such weakened organisms, while losing agricultural value, may exhibit narrow resistance to certain types of viruses [9,19,20]. At the same time, the appearance of even a single compensatory amino acid substitution in VPg may be sufficient to restore its ability to form a complex with eIF4E, making the plant susceptible to viral infection once again [21,22,23]. One of the most promising alternative strategies for combating infections caused by this virus family is the introduction of targeted mutations in specific host protein genes to alter their functional properties [24,25,26,27]. This may allow, while preserving the core function of the protein in the cell, for its ability to interact with viral components to be completely disabled or severely impaired on a long-term basis. The search for such mutations is possible through the rational design of specific amino acid substitutions using an integrated approach that includes both various bioinformatic methods and in vitro experiments. Partial application of this approach has already proven effective; using it, melon plants resistant to MWMV [28] and watermelon plants resistant to ZYMV, PVY, and PRSV were obtained [13].

For some time, it was believed that the VPg protein of potyviruses is unstructured in its free state. However, relatively recently, the structure of VPg from *Potato virus Y* was determined by NMR, revealing the presence of a globular domain in the protein [29].

To date, there is no consensus on the role of the VPg–eIF4E complex in the viral life cycle. Some researchers believe that this complex is necessary to initiate the translation of viral RNA, as VPg mimics the cap [29,30,31]. Other scientists propose that potyviruses employ an alternative mechanism of translation initiation, in which the 5′-UTR acts as an internal ribosome entry site (IRES), and the formation of the eIF4E–VPg complex is instead required to suppress cap-dependent translation of host cellular mRNA [32,33,34]. In studies of the interaction between eIF4E and eIF4G in melon, specific motifs on both proteins involved in complex formation were identified. Due to the similarity between one region of the VPg sequence and a motif on eIF4G that interacts with eIF4E, researchers have speculated that VPg competes with eIF4G for binding to eIF4E [35,36]. Thus, the possible mechanisms of functional VPg activity can be divided into three groups: (I) VPg mimics the cap, interacts with eIF4E in the cap-binding pocket and, as a result, thereby competes with host cap-mRNA, apparently initiating viral mRNA translation, (II) VPg competes for the binding of eIF4E to eIF4G, suppressing the translation of cellular mRNA, and (III) VPg interacts with eIF4E either in the cap-binding pocket or at the eIF4G binding site, combining features of both mechanisms above. The first mechanism of VPg–eIF4E complex formation appears to be the most plausible, as it best aligns with available biochemical data. However, just as there is no consensus on the mechanism of complex formation, there is also no agreement on the specific roles of key amino acid residues in forming the intermolecular interface. These intermolecular contacts are critical for complex formation and, consequently, for the rational design of mutations that can prevent this interaction on a long-term basis.

Even in the absence of direct experimental data on the spatial organization of molecules in the VPg–eIF4E complex, the interpretation of available biochemical data from a structural perspective is possible using methods of molecular modelling and molecular dynamics. To date, several models of VPg complexes from various representatives of the Potyviridae family with host plant eIF4E have been constructed using molecular modelling [29,37]. These models are based on the assumption that VPg interacts with eIF4E in the cap-binding pocket. We have also proposed our own version of this complex, developed through the analysis of available structural data, results from yeast two-hybrid assays, and multiple sequence alignment of VPg from PVY [38,39]. According to our model, VPg interacts with the α-hairpin adjacent to the cap-binding pocket of eIF4E in such a way that the conserved residue R104, located on this hairpin, forms the primary recognition contact during the formation of the eIF4E–VPg complex. Although this model aligns well with available biochemical data, it remains a theoretical construct and requires further refinement.

Research in the field of potyvirus phylogenetics is aimed at identifying mutations associated with biological traits, assessing the rate of mutation emergence and fixation, and reconstructing evolutionary trees to determine the origin and assess the prevalence of potyviruses. Phylogenetic and recombination analyses of complete PVY sequences have made it possible to classify the numerous strains of this virus into several phylogroups: the O group, N group, C group, and the recombinant groups R1 and R2 [4,5,8]. Molecular dating has shown that the introduction of potato virus Y coincided with the period during which potatoes were imported into Europe [40]. In addition to analyzing complete sequences, studying small fragments of genomic RNA is also important for identifying functionally significant regions within protein-coding sequences. For example, plant-specific and strain-specific mutations have been identified in the coat protein (CP) of potato virus Y [41,42]. Phylogenetic analysis of VPg PVY sequences has revealed positive selection in a region of the sequence that is presumably responsible for forming the eIF4E binding site [20].

According to the law of homologous series [43], the mechanism of interaction between eIF4E and VPg identified in one species should also be characteristic of all species within this family, provided that taxonomic units are formed based on genetic principles. Through multiple sequence alignment, it is possible to determine which regions of the sequence are conserved or variable and to identify mutations associated with biological traits. Conserved residues are most likely to play key roles, as they are typically involved in maintaining specific three-dimensional structures and/or in forming critical intermolecular contacts. In this article, we updated and expanded the frequency analysis of amino acid sequences for VPg PVY and performed a multiple alignment of VPg sequences from several Potyviridae species. This was done to identify patterns that support or contradict our model of the interaction between VPg and proteins of the eIF4E family. We found arginine residues in VPg of different species of each genus within the Potyviridae family that are invariant and occupy position in the hairpin like an arginene of VPg PVY.

## 2. Results and Discussion

### 2.1. Multiple Sequence Alignment VPg PVY

All analyzed sequences encoding VPg PVY were combined into several groups (Table 1). Selection group № 1 is the largest and includes all sequences encoding VPg PVY. Based on this dataset, additional subsets were formed: selection group № 2 (572 sequences belonging to the NTN phylogroup), selection group № 3 (512 sequences from the O phylogroup), selection group № 4 (sequences from the C phylogroup), and selection group № 5 (92 sequences from the N phylogroup). Further grouping was performed based on host plant origin. From selection group № 2 and № 3, subsets № 6 and № 7 (from selection group № 2), and № 8 and № 9 (from selection group № 3) were created, grouping sequences according to the plant species from which the corresponding isolates were collected.

Multiple alignment of VPg PVY sequences allows for the identification of the most prevalent strains. The analysis revealed that potato virus Y is widespread globally. The most common strains belong to the O and NTN phylogroups. These findings are consistent with those reported by other researchers [3,4,8]. The highest number of isolates was obtained from potato and tobacco plants, likely reflecting the economic importance of these crops. Among potato-derived isolates, the most common strains are from the O and NTN phylogroups. Based on these observations, these phylogroups became the focus of our further analysis.

### 2.2. Analysis of Protein VPg PVY Conservation from a Structural Perspective

**Figure 1:**
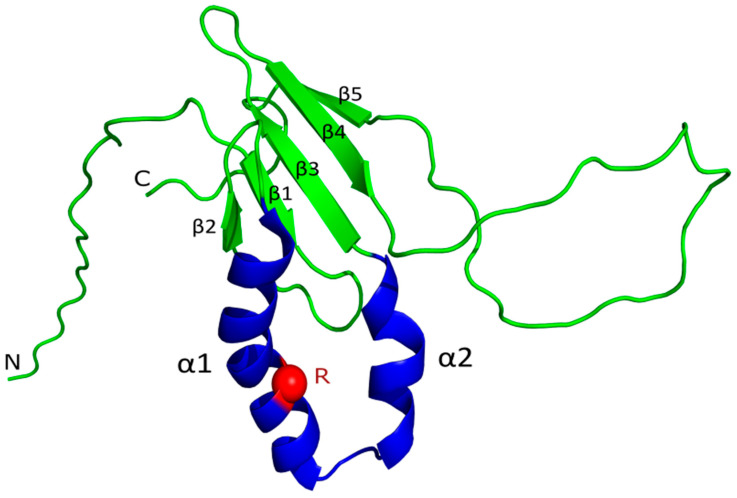
VPg PVY protein NMR structure (PDB ID 6NFW). Full model are colored in green. The α1–α2 hairpin is highlighted in blue. Major structural elements are labeled. The red sphere indicates arginine at position 104, which is the main object of interest in this article.

According to currently available experimental structural data, VPg from PVY consists of a central structured domain and unstructured N- and C-terminal regions. The central domain is composed of a β-sheet formed by five antiparallel β-strands, an α1–α2 hairpin, and an extended β4–β5 loop (Figure 1). Notably, according to the NMR structure, the α1–α2 helix has a distorted conformation and is relatively labile.

**Figure 2:**
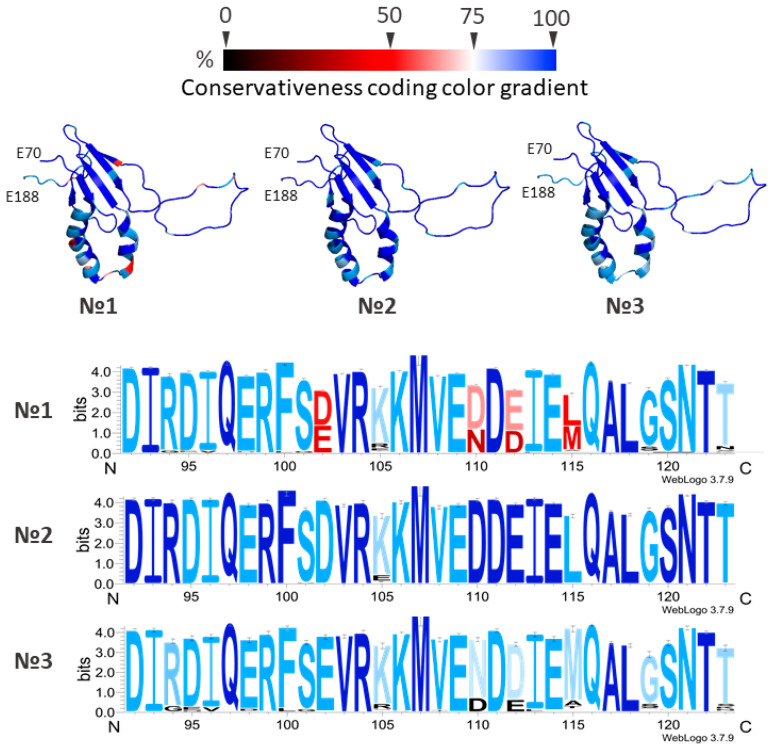
Structure of the VPg PVY (residues 70–188) and amino acid profiles of the α1–α2 hairpin region (residues 92–123 aa). Colouring reflects the conservation level of amino acid residues, with the conservation gradient scale shown at the top: values from 0% (fully variable amino acid residues) to 100% (invariant acid residues). Three VPg models (70–188 aa) are presented under the gradient. The coloring of each of the three models corresponds to the frequency of amino acids found in the selection group. The numbers (№) correspond to the selection group described in the text. Three amino acid profiles (92–123 aa) are presented under the models. The height of the symbols in the logos reflects the frequency of the amino acid in each position in bits. The coloring of the amino acid profiles performed with the help of the same gradient scale and corresponds to the frequency of amino acids.

Of particular interest is the α1–α2 hairpin sequence (amino acids 92–123, numbered according to accession KC005979), as this region is believed—based on current theoretical models and biochemical data—to participate in the formation of the VPg•eIF4E complex [38,39]. Frequency analysis of selection group № 1 of the VPg PVY amino acid sequences revealed the most common substitutions in the α1–α2 hairpin region, occurring at positions 102, 110, 112, and 115 (Figure 2).

The identified positions with variable amino acids reflect differences between the two most common phylogenetic groups, which is clearly visible in the amino acid frequency profile of the α1–α2 hairpin region (Figure 2). We speculate that substitutions at positions 96, 101, 105, and 119 appear to be adaptive in nature. These residues likely modify the α1–α2 hairpin to better accommodate interaction with the eIF4E cap-binding pocket. Such substitutions probably do not significantly alter or destabilize the overall protein structure, as structurally disruptive changes would likely be eliminated through natural selection—or, conversely, a structurally distinct new form of the protein would become dominant. For the most common mutant forms, structural models were generated using AlphaFold3. However, this method does not yet allow for reliable assessment of the structural impact of single amino acid substitutions [44].

**Figure 3:**
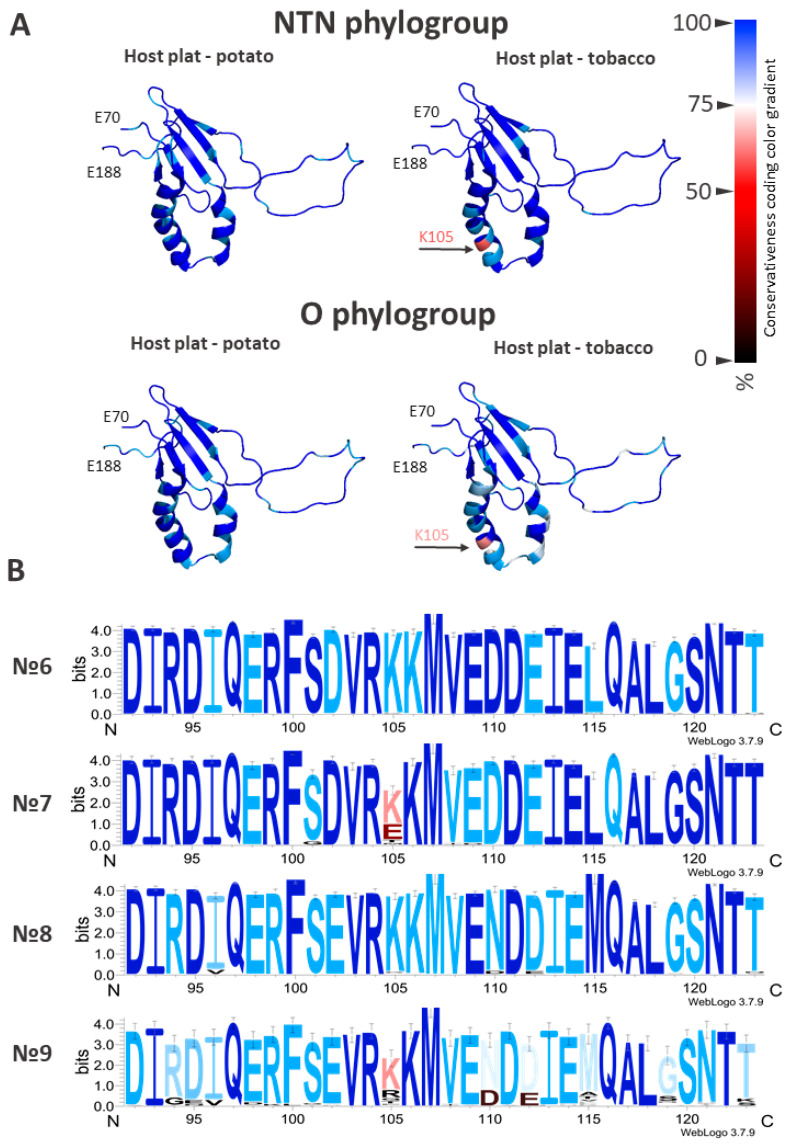
Structure of the VPg PVY and amino acid frequency profiles of the α1–α2 hairpin region (residues 92–123). Colouring reflects the conservation level of amino acid residues, with the conservation gradient scale shown at the right: values from 0% (fully variable amino acid residues) to 100% (invariant acid residues). (**A**) Four VPg models (70–188 aa) are presented. The color of each of the four models corresponds to the frequency of amino acids found in the selection group. Numbers (№) correspond to the sample groups described in the text. (**B**) Four amino acid profiles (92–123 aa) are presented under the models. The height of the symbols in the logos reflects the frequency of the amino acid in each position in bits. The coloring of the amino acid profiles performed with the help of the same gradient scale and corresponds to the frequency of amino acids.

Interestingly, the long disordered N-terminal region is relatively conserved based on multiple alignment of complete (full length) sequences. The most frequent substitutions in this region occur at positions 57, 59, and 61. It is possible that this region adopts a defined structure upon interaction with one of its partner proteins (e.g., eIF4E, HC-Pro, etc.). Selection group № 6 includes 312 sequences, all annotated with potato as the host plant. Of these, 258 sequences (82%) are identical. This consensus sequence is considered the representative or reference form for the NTN phylogroup. The remaining 54 sequences contain amino acid substitutions. Frequency analysis of the multiple alignment revealed several recurring mutations, particularly in the α1–α2 hairpin region, including K105R, L115M, and G119S. However, these substitutions do not significantly alter the overall amino acid profile in this region (Figure 3). Overall, selection group № 6 can be characterized as highly conserved.

The following conservative positions are present in the selection group № 1: I93, Q97, V103, R104, M106, D110, A116, L117, and T122. These positions are typical for all sequences, regardless of the strain. This may indicate their importance. These positions could perform a structural function, supporting the three-dimensional structure of the protein, or directly participate in the recognition of the partner molecule. Among the conserved amino acid residues listed, arginine can mimic the cap structure in the best way [38]. We consider this amino acid residue to be key for the formation of specific bonds with the partner protein eIF4E. The role of other conserved amino acid residues can be guessed from the intermolecular structure context. Obviously, at least some atoms of a residue must form the surface of the protein globule to have a chance to participate in the intermolecular interface formation. The side groups of amino acid residues that are exposed to the solvent are most likely candidates for this. If the side chain of an amino acid is oriented in a way that allows it to form an intramolecular hydrogen bonds or other intramolecular interactions, these positions are likely to play an important role in maintaining the three-dimensional structure of the molecule.

Selection group № 7 (isolates collected from tobacco) includes 177 sequences, 77 of which correspond to the base type—representing 43% of the sample. Thus, substitutions in NTN strain sequences are considerably more common among isolates collected from tobacco plants than those from potato plants. Within the α1–α2 hairpin, substitutions were found at positions 101 and 105, which deserve particular attention. These occur more frequently than other substitutions and are typically non-homologous (Figure 3).

A similar frequency analysis was performed for selection group № 8. Of the 332 sequences from phylogroup O isolates collected from potato plants, 183 (55% of the sample) correspond to the base type. The remaining sequences contain substitutions that are relatively evenly distributed along the protein. Several substitutions are localized in the α1–α2 hairpin at positions 96, 105, 110, 112, 119, and 123. However, these substitutions do not significantly affect the overall amino acid frequency profile (Figure 3).

Frequency analysis of selection group № 9 (phylogroup O isolates collected from tobacco plants) revealed that 32 sequences (48%) correspond to the base type. The remaining 34 sequences contain various substitutions, most of which are concentrated in the α1–α2 hairpin region (Figure 3).

Phylogroups C (selection group № 4) and N (selection group № 5) were the least represented. For these groups, amino acid frequency profiles of the α1–α2 hairpin are shown in Supplementary Figure S1. This limited representation may be due to the low virulence or restricted distribution of the strains comprising these phylogroups. A comprehensive list of common substitutions identified in each sample is presented in Supplementary Table S1.

Isolate sequences included in selection group № 5 are predominantly represented by the basic type. Most of the observed substitutions in this group are located in the β-sheet region and rarely occur in the α1–α2 hairpin (Figure 3). In contrast, sequences in selection group № 6 are also mainly of the basic type, but in this case, most substitutions are concentrated in the α1–α2 region (Figure 3). A clear correlation was observed between substitutions at position 105 in the α1–α2 hairpin and the host plant—tobacco (Fisher’s exact test, *p* < 0.05). The lysine was the predominant residue at position 105 in both host groups, it was significantly more frequent in potato-derived isolates (98.1%, *p* < 0.05) than in tobacco-derived isolates (63.7%, *p* < 0.05). For VPgs from potato-derived isolates, there is a strict limitation on the presence of positively charged amino acids at this position. In contrast, there is no such limitation on isolates collected from tobacco plants. Interestingly, E105 (22.36%, *p* < 0.05), M105 (2.95%, *p* < 0.05), R105 (4.64%, *p* < 0.05) and T105 (6.33%, *p* < 0.05) were found exclusively or predominantly in VPgs from tobacco-derived isolates, while they were completely absent from potato-derived isolates (*p* < 0.05 for each comparison). These substitutions meet several statistically significant criteria simultaneously: high frequency of occurrence, correlation with a biological trait, and localization in a functionally important region. The K105R substitution is extremely rare (1.9%) among isolates collected from potato plants and does not significantly affect the overall amino acid frequency profile in this group.

Additionally, the substitution at position 101, which appears in selection groups № 7, № 8, and № 9, may contribute to the formation of a more stable VPg•eIF4E complex. The potential effect of this substitution was discussed in our previous work [39].

### 2.3. Multiple Alignment VPg of Potyviridae Family Representatives

By comparing the sequences of VPg representatives of different genera of the family of potyviruses, it is possible to identify conserved amino acid residues occupying the same positions in space. They are likely to play either an important structural role or be directly necessary for the functional activity of the protein [45,46].

**Figure 4:**
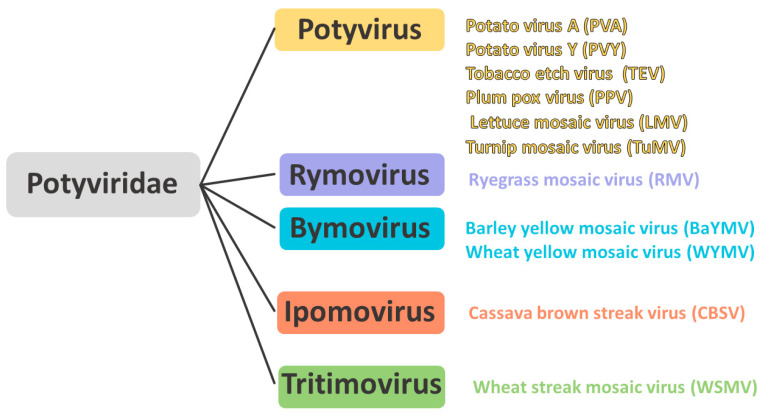
Potyviridae family representatives studied in this research.

The most common widespread representatives of each genus within the Potyviridae family were selected for analysis. Multiple alignment of nucleotide sequences encoding the VPg was performed for potato virus Y (PVY; 1197 sequences), potato virus A (PVA; 85 sequences), tobacco etch virus (TEV; 59 sequences), lettuce mosaic virus (LMV; 120 sequences), turnip mosaic virus (TuMV; 1135 sequences), plum pox virus (PPV; 633 sequences) from the genus Potyvirus, ryegrass mosaic virus (RMV; 10 sequences) from the genus Rymovirus, barley yellow mosaic virus (BaYMV; 83 sequences) and wheat yellow mosaic virus (WYMV; 159 sequences) from the genus Bymovirus, wheat streak mosaic virus (WSMV; 111 sequences) from the genus Tritimovirus, and Cassava brown virus (CBSV; 72 sequences) from the genus Ipomovirus (Figure 4).

The most common representatives of each genus within the Potyviridae family were selected for analysis. Nucleotide sequences were translated to produce amino acid sequences. As a result of multiple sequence alignments (MSAs), consensus sequences were generated for each species. It should be noted that virus types are represented by varying numbers of sequences—the more widespread and well-studied species, such as PVY or PPV, are associated with larger sample sizes. A small number of aligned sequences leads to an increase in the probability of error, which is reflected in the growth of error bars (Figure S2).

### 2.4. Obtaining and Comparing Models of Members of the Potyviridae Family

**Figure 5:**
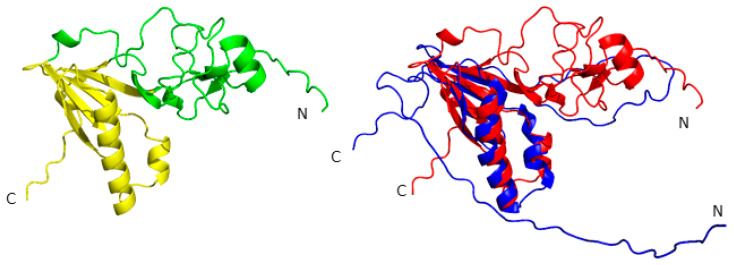
Cartoon presentation of the VPg PVY model. On the left: the consensus VPg PVY model obtained using Alphafold3. The central domain is highlighted in yellow; the N-terminal domain is highlighted in green. On the right: structural comparison between the NMR structure of VPg PVY (fragment 73–188 aa) shown in blue, and the AlphaFold3-generated model of the same protein shown in red.

Using AlphaFold3 and the consensus amino acid sequences for each of the above-mentioned Potyviridae species, corresponding consensus VPg models were constructed. All models display a similar structural organization of the central domain, which is composed of an antiparallel β-sheet, an extended β4–β5 loop, and an α1–α2 hairpin (Figure 5). However, some of the resulting VPg models comprise two domains. One of these, as in the VPg PVY NMR structure, corresponds to the central domain. The second domain is formed from the N-terminal region—previously considered unstructured based on NMR data—and the β4–β5 loop, which together form a stable tertiary structure (according to molecular dynamics results).

It is worth noting that many models generated using the updated AlphaFold3 algorithm differ significantly from those created with the earlier AlphaFold2 version. Previously, we obtained only the central domain in globular form, consistent with available experimental structural data (PDB ID: 6NFW). In contrast, the AlphaFold3-generated models fold the entire amino acid sequence into a globular structure. As noted, the new models exhibit a dual-domain architecture and lack extended unstructured regions.

According to the predicted local distance difference test (pLDDT), the confidence level of these new models is relatively high. Our molecular dynamics simulations support the stability of these structures: over a 400 ns trajectory, the overall fold of the protein’s Cα backbone was preserved, although some loop regions exhibited high flexibility. Nonetheless, there is currently no experimental evidence confirming the existence of a VPg protein with this type of spatial organization in solution.

According to available data, the α1–α2 hairpin plays an important role in the formation of the complex between eIF4E and VPg [29,39]. Molecular modelling results showed that this hairpin is present in all analyzed representatives from different genera of the Potyviridae family, although its length varies. Although the species are part of the same family, their full-length protein sequences exhibit a low degree of similarity to the VPg PVY strain NTN: PVA—50.5%; TEV—51.4%; PPV—54.1%; TuMV—50.78%; LMV—47.13%; RMV—36.0%; WSMV—26.6%; WYMV—31.1%; BaYMV—31.9%; CBSV—26.45%. The α1–α2 hairpin sequences show a low percentage of identity relative to the VPg strain PVY NTN: PVA—23.7%; TEV—26.7%; PPV—20.0%; TuMV—20.0%; LMV—10.0%; RMV—36.0%; WSMV—10.0%; WYMV—31.1%; BaYMV—31.9%; CBSV—26.45%. Despite the low values of the identity in the amino acid sequence of the regions corresponding to the α1–α2 hairpin, this region has an almost the same conformation and a high pLDDT value. Analysis of amino acid profiles allows for the localization of conserved residues within the α-hairpin sequence but does not provide information about their relative spatial positions (Figure S2).

**Figure 6:**
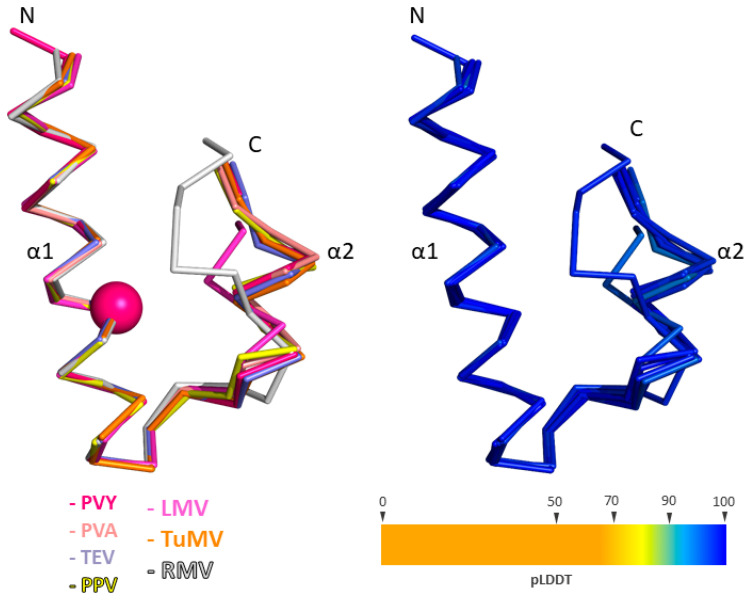
Cα-models of VPg PVY, PVA, TEV, PPV, LMV, TuMV, and RMV hairpins. On the left side spheres indicate the positions of the Cα atoms of the recognizing arginine residues. Color coding is shown in the image. On the right side the Cα atoms are colored according to the pLDDT (predicted Local Distance Difference Test) value.

According to the theoretical model of the complex previously developed by our group, the key recognition contact on the part of VPg is formed by the side chain of an invariant arginine at position 104 [38,39]. The α-hairpin region, overall, appears to be highly conserved in structure. As a result of molecular modelling, the amino acid sequences corresponding to this structural element in PPV, PVA, TEV, TuMV, and PVY fold into nearly identical spatial conformations. The maximum relative displacement of corresponding Cα atoms in the α1 helix and apical loop regions averaged around 0.4 Å, while in the more flexible α2 helix region, the displacement was approximately 1.2 Å. For LMV and RMV, the α1 helix and apical loop regions showed a similar degree of structural conservation; however, the position and conformation of the α2 helix differed more significantly. In RMV, the α2 region does not form a helical structure at all. Nonetheless, the spatial positioning of the recognizing arginine residue remained consistent among these viruses (Figure 6).

In VPg of BaYMV, WYMV, WSMV, and CBSV viruses, phylogenetically removed from the genera Potyvirus and Rymovirus, the Cα atom of arginine, presumably having a recognizable function, occupies different positions within the hairpin. Despite this, the functional group of its side chain, responsible for forming the recognition contact, may still adopt the appropriate spatial position.

According to the amino acid frequency profile, there is at least one invariant arginine for the α1–α2 hairpin region: PVY—R104; PVA—R103; TEV—R102; PPV—R103; LMV—R96; TuMV—R103; RMV—R106; BaYMV—R98; WYMV—R112; WSMV—R123; CBSV—R117 (Figure S2). This position is subject to significant purifying (negative) selection. According to our model, this position is conservative, since arginine plays an important structural role in the formation of the recognition contact with glutamate on the eIF4E side. Recognizing contact is most important for the formation of a complex. The remaining amino acid residues in the α1–α2 hairpin will form stabilizing contacts with the surface of the cap-binding pocket.

A comparison of amino acid profiles and VPg models of different representatives of the Potyviridae family allows us to note the similarity of some conservative positions. For several VPgs (VPg PVA, VPg TEV, VPg PPV. VPg TuMV, VPg CBSV) there is also an I at the base of the hairpin, as in I93 VPg PVY. Conservative M (M106 VPg PVY) is indeed found in many of the α1–α2 hairpins of VPg from different species of the Potyviridae family. However, its position in different α1–α2 hairpin varies greatly, which is most likely due to the adaptation of a specific hairpin to a particular eIF4E cap-binding pocket. The presence of D (D110 VPg PVY) in the bridge between α1 and α2 is also characteristic of many of the representatives considered. This amino acid occupies a different position depending on the representative. The function of this conserved amino acid can be attributed to the need to form a bond with the positively charged surface of the eIF4E cap-binding pocket. As for the remaining conservative positions A116, L117, and T122 in the α1–α2 hairpin VPg PVY, no similar conservative positions were found in the α1–α2 hairpins VPg from different species of the Potyviridae family in similar spatial positions. So, it is difficult to guess their roles.

### 2.5. The Results of Molecular Dynamics Experiments

Molecular dynamics experiments showed that the R104 side chain in the theoretical consensus model remains solvent-exposed throughout the simulation trajectory, making it accessible for potential interaction with the recognition site on eIF4E. In the only known VPg PVY NMR structure, this R104 residue is also solvent-exposed in all 10 presented models. Moreover, MD simulations using the experimental structure as the starting model confirmed that this positioning of R104—allowing it to function as a key recognition element—is maintained (one of the trajectory is available via the link https://doi.org/10.5281/zenodo.18045050).

Similar molecular dynamics trajectories were calculated for VPg from representatives of various genera within the Potyviridae family. These simulations demonstrated that, in the α-hairpin region of all VPg analyzed, an arginine side chain remains exposed and capable of forming a recognition contact—similar to the interaction proposed in our previously developed VPg•eIF4E complex model [39].

### 2.6. Role of Mutations in Adaptation to the Host Plant

**Figure 7:**
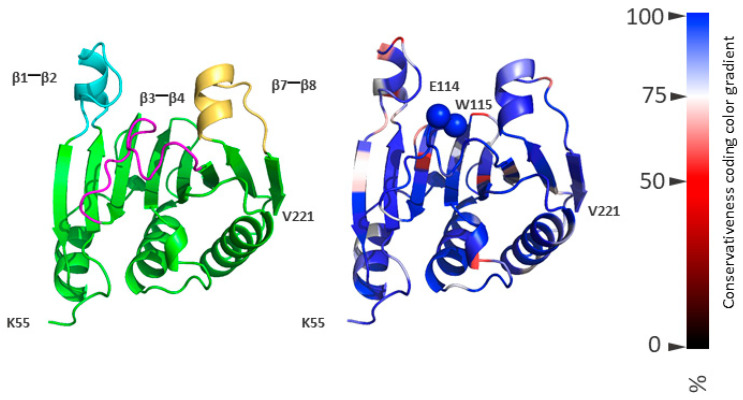
eIF4E model of *Solanum tuberosum*. (**Left**) Loops β1–β2, β3–β4, and β7–β8, forming the walls of the cap-binding pocket, are coloured in cyan, magenta, and wheat, respectively. (**Right**) Cα atoms of residues W114 and E115 are shown as spheres. Colouring reflects the conservation level of amino acid residues, with the conservation gradient scale shown at the right: values from 0% (fully variable amino acid residues) to 100% (invariant acid residues).

In eukaryotes, the cap-dependent mechanism of translation initiation is the primary pathway. eIF4E plays a central role in this process by interacting with both the mRNA cap and eIF4G54 during the formation of the initiator complex. In most organisms, eIF4E is represented by a family of proteins [12,47]. The walls of the cap-binding pocket of eIF4E are formed by three loops: β1-β2, β3-β4, and β7-β8, while the base of the pocket is formed by an antiparallel β-sheet (Figure 7). A substantial number of eIF4E homologue structures from different organisms have been characterized. These homologues show a high degree of conservation not only in their primary sequences but also in their tertiary structures. The cap-recognition module is formed by the highly conserved β3–β4 loop. Within this loop, invariant amino acid residues—specifically W114 and E115 (numbering according to MT828873)—are involved in forming recognition contacts. These residues are implicated in interactions with the cap structure of mRNA (based on structural data) and, according to our model, also play a key role in the interaction with VPg. The cap-recognition module is formed by the highly conserved β3–β4 loop. Within this loop, invariant amino acid residues—specifically W114 and E115 (numbering according to MT828873)—are involved in forming recognition contacts. These residues are implicated in interactions with the cap structure of mRNA (based on structural data) and, according to our model, also play a key role in the interaction with VPg.

Such an unchanged structure of a highly selective recognition module across distantly related eukaryotic species suggests an extremely limited set of possible spatial configurations for the corresponding ligand-binding region. Accordingly, the recognition region of the VPg structure must consistently position its functional groups in space to ensure the formation of specific intermolecular contacts. This implies a high degree of conservation—or even invariance—of the amino acids carrying these functional groups.

**Figure 8:**
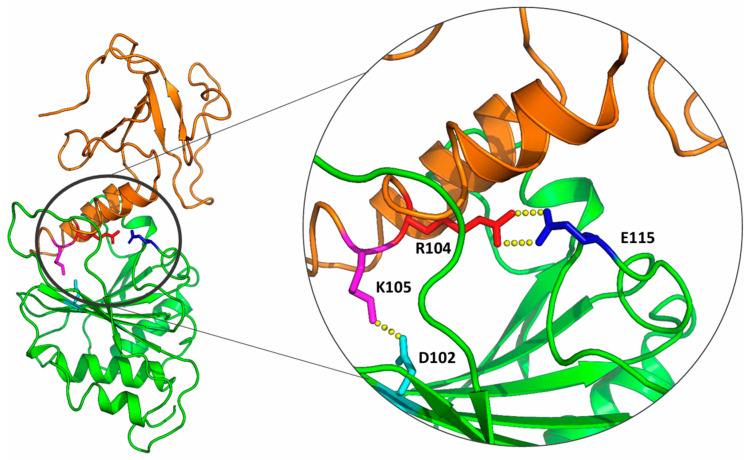
The model of the VPg•eIF4E complex. VPg from PVY is shown in orange; *Solanum tuberosum* eIF4E is shown in green. The amino acid residues R104 (VPg) and E115 (eIF4E), which form a key recognition contact, are highlighted in red and blue, respectively. Residues K105 (VPg) and D102 (eIF4E), which form an additional stabilizing contact, are shown in magenta and cyan, respectively. Hydrogen bonds are shown in yellow.

In this context, while the overall structure of eIF4E is conserved, the network of stabilizing contacts within different plant eIF4E homologs may vary. As a result, the corresponding interface regions of VPg are likely adapted to the specific eIF4E variant of the host plant. According to our model of the VPg•eIF4E complex, the NZ atom of K105 in VPg lies within hydrogen-bonding distance of the OE1 and/or OE2 atoms of D102 in eIF4E. These residues are located at the base of the cap-binding pocket (Figure 8). Position 102 in eIF4E is generally conserved; however, rare non-homologous substitutions such as D102N or D102G can occur. These substitutions may be functionally tolerated if accompanied by compensatory changes in nearby residues, allowing the recognition contact to be preserved.

## 3. Materials and Methods

### 3.1. Multiple Sequence Alignments

VPg nucleotide sequences of several species of various genera within the Potyviridae family (PVY, PVA, TEV, PPV, LMV, TuMV, RMV, BaYMV, WSWV, CBSV, and WYMV) were retrieved from the NCBI database for alignment. Multiple sequence alignment was performed using MUSCLE (MUltiple Sequence Comparison by Log-Expectation) implemented in the Unipro UGENE software package v50.0 64-bit version Apr 11 2024 [48,49,50]. Nucleotide sequences were translated into amino acid sequences using the same software.

VPg PVY sequences were grouped into four phylogroups: O, C, NTN, and N. The genomic RNA region encoding VPg contains the recombination point between the O and N strains. This allows differentiation between the O, N, and NTN (recombinant) strains without requiring phylogenetic analysis of the full genome. All recombinant strains, including NTNa, NTNb, SYR-I, SYR-II, SYR-III, and E, were grouped into a single NTN phylogroup. Similarly, strains O, O5, N:O, and N-Wi were grouped into the O phylogroup.

Since differences between strains within the same phylogenetic group cannot be determined by analyzing only a small fragment, full-genome sequence analysis is necessary. Sequence affiliation to a specific strain was reassessed using reference nucleotide sequences [8,51], regardless of the strain indicated in the original annotation. This was necessary due to the presence of numerous annotation errors, which arose because independent phylogenetic data were not available at the time the viral cDNA sequences were published. Based on the alignments, conservation and amino acid substitutions were assessed. The complete VPg PVY protein sequence consists of 188 amino acid residues across all members of the species. Incomplete sequences (shorter than 188 amino acids) were excluded from further analysis. Multiple alignments were also performed for VPg sequences from the other Potyviridae species considered in this study; however, no division into phylogroups and strains was made for these.

A list of all accession numbers for the sequences analyzed in this study is provided in the Supplementary Materials (Table S1).

### 3.2. Molecular Modelling and Molecular Dynamics

Consensus VPg models for different representatives of the Potyviridae family were created using consensus amino acid sequences for each species and the AlphaFold3 algorithm [52]. Of the five models generated by the algorithm, model 0 (with the highest pLDDT value) was selected for further refinement.

The geometry of each selected model was refined using the Phenix software version 1.21.1-5286-000. Manual editing of the models was performed using the COOT software package v0.9.8.96. Ramachandran maps and statistics for bond lengths and angles for each start model are presented in the Supplementary Materials. System parameters for the molecular dynamics (MD) simulations were set using the CHARMM-GUI web interface. A cubic simulation box measuring 100 × 100 × 100 Å was used, ensuring a minimum distance of 10 Å between the protein and the box boundaries. Solvation was carried out using the TIP3P three-point water model. Na^+^ and Cl^−^ ions were added to achieve a physiological salt concentration of 150 mM. Molecular dynamics simulations were performed using the GROMACS 2023.5 software package. Energy minimization of the system was conducted using the steepest descent algorithm until the maximum force reached 500 kJ∙mol^−1^∙nm^−1^. Temperature equilibration was carried out in the NVT ensemble at 310 K for 1000 ps, using a 2 fs integration step and the V-rescale thermostat algorithm. Pressure equilibration followed in the NPT ensemble at 1 Pa using the same time parameters and the C-rescale barostatting algorithm. Long production MD simulations were run in the NPT ensemble for 400 ns using the Parrinello–Rahman barostat and the all-atom Charmm36 force field.

### 3.3. Data Analysis and Visualization

The WebLogo tool (https://weblogo.threeplusone.com/ was used to generate amino acid profiles [53]. The ordinate scale is indicated in bits and corresponds to the information content for each alignment position. Error bars represent the standard deviation, and each position is color-coded according to the frequency of amino acid occurrence.

Superposition and comparative analysis of experimental and theoretical eIF4E models were performed using the COOT and PyMOL software packages (PyMOL Version 2.6.2, OPEN-SOURCE). Conservation-based coloring of models derived from multiple sequence alignments was done in PyMOL using a custom script created using DeepSeek (https://github.com/Victoria-Kolesnikova/-coloring-reflects-the-conservation-level.git, accessed on 25 January 2026).

Analysis and visualization of the MD trajectories were carried out using VMD software v.2.0.0a7.

## 4. Conclusions

Analysis of the multiple alignment of VPg PVY amino acid sequences revealed a number of common substitutions in the α1–α2 hairpin region. The detected substitutions can be divided into two categories: strain-specific and adaptive. Strain-specific substitutions are generally homologous and do not affect the functional activity of the protein. Adaptive substitutions—such as those at positions 101 and 105—modify the binding interface to facilitate stable VPg complex formation with a specific eIF4E variant from a particular host plant. Substitutions in position K105 are most often observed in isolates collected from tobacco plants. Perhaps they can be both adaptive and plant-specific.

Conserved amino acid residues generally serve important structural or functional roles. Invariant residues found within the α1–α2 hairpin of VPg PVY may be capable of forming recognition contacts. According to our model, the invariant arginine at position 104 is one such residue, capable of establishing a key recognition contact. Analysis of the multiple alignment of VPg amino acid sequences from representatives of different genera within the Potyviridae family showed that each sampled species contains at least one invariant arginine within the α1–α2 hairpin. Comparative structural analysis of theoretical VPg models from these genera demonstrated that, at the interface with eIF4E, the side chain of this arginine occupies a position suitable for forming hydrogen bonds with residue E115 of eIF4E. These findings are consistent with our earlier results and support the interaction model we previously proposed.

## Figures and Tables

**Table 1:** Selection groups of sequences encoding VPg PVY.

	Phylogroup	Host Plant Selection Group	Sequences Count
VPg *Potato virus Y* № 1	NTN group (572) № 2	*Solanum tuberosum* № 6	312
		*Nicotiana tabacum* № 7	177
		Other	83
	O group (512) № 3	*Solanum tuberosum* № 8	332
		*Nicotiana tabacum* № 9	66
		Other	114
	C group (15) № 4	Other	
	N group (92) № 5	Other	

## Data Availability

The original contributions presented in this study are included in the article/supplementary material. Further inquiries can be directed to the corresponding author(s).

## Supplementary Materials

The following supporting information can be downloaded at: https://www.mdpi.com/article/10.3390/ijms27073280/s1, Table S1—https://doi.org/10.5281/zenodo.16787720. The 400 ns molecular dynamic (MD) trajectories for the wild type VPg and statistical data on the obtained models: https://doi.org/10.5281/zenodo.18951896.
