# Supplementary material for: Conserved Arginine of the Potyviridae Viral Genome-Linked Proteins (VPg) as a Key Determinant for eIF4E Binding
Source: Int J Mol Sci. 2026 Apr 4;27(7):3280. doi: 10.3390/ijms27073280 (PMC13073118; doi:10.3390/ijms27073280)
Supplement: Supplementary file 1 [file ijms-27-03280-s001.zip › ijms-4146413-supplementary.pdf]

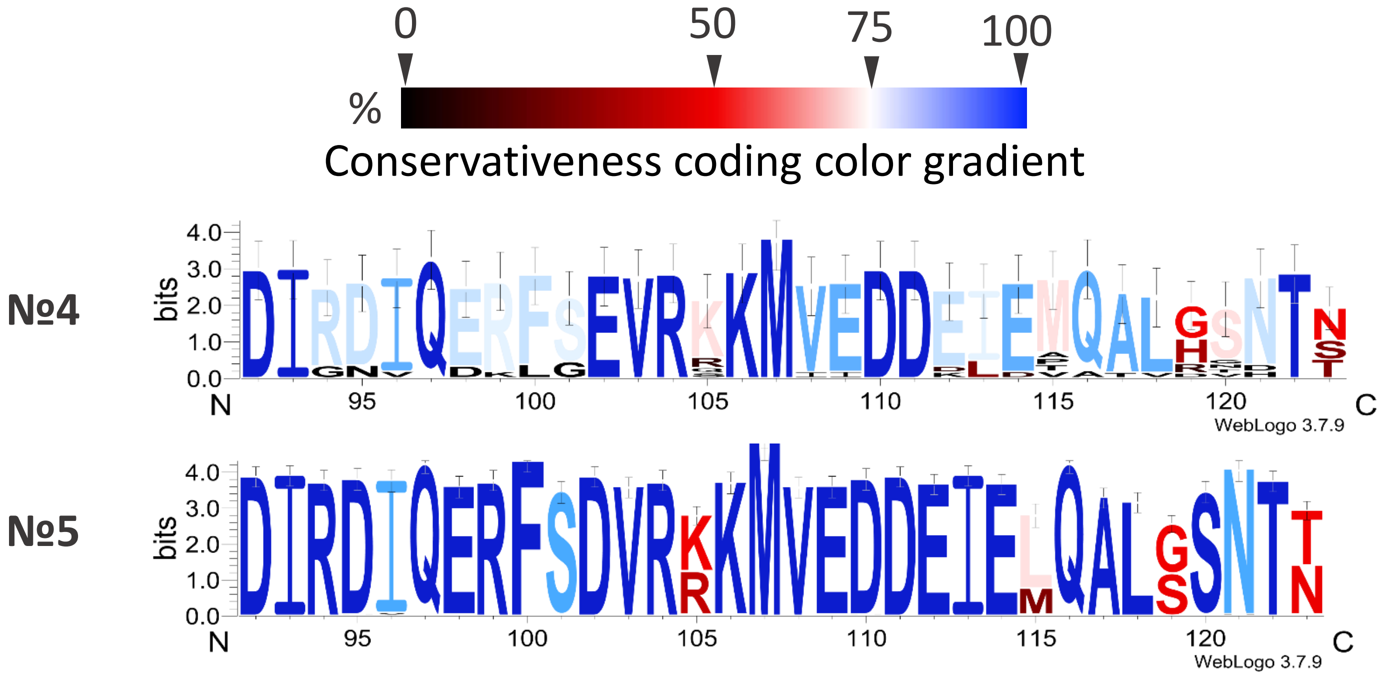

Figure S1.

Amino acid frequency profiles of the α1–α2 hairpin region (residues 92–123) VPg PVY. Colouring reflects the conservation level of amino acid residues, with the conservation gradient scale shown at the top. Numbers (№) correspond to the sample groups described in the text.

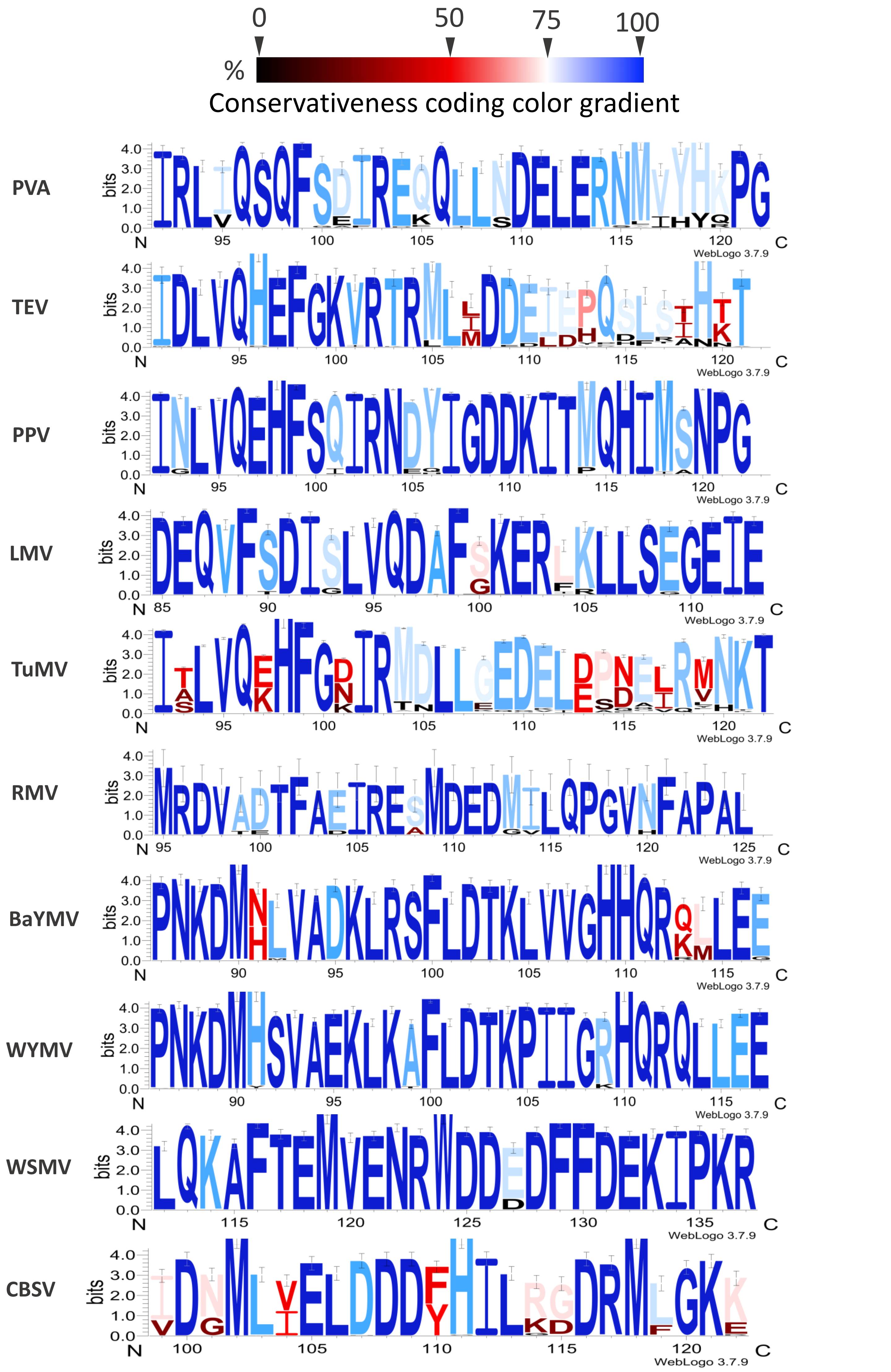

Figure S2.
Amino acid frequency profiles of the α1–α2 hairpin region a few representatives of different genera of *Potyviridae* family . Colouring reflects the conservation level of amino acid residues, with the conservation gradient scale shown at the top.
